# Activity of Silver Nanoparticles against *Staphylococcus* spp.

**DOI:** 10.3390/ijms23084298

**Published:** 2022-04-13

**Authors:** Denis Swolana, Robert D. Wojtyczka

**Affiliations:** Department of Microbiology and Virology, Faculty of Pharmaceutical Sciences in Sosnowiec, Medical University of Silesia in Katowice, ul. Jagiellońska 4, 41-200 Sosnowiec, Poland; dswolana@sum.edu.pl

**Keywords:** silver nanoparticles, *Staphylococcus epidermidis*, *Staphylococcus aureus*, antimicrobial activity, antibiofilm activity

## Abstract

*Staphylococcus epidermidis* is a bacterium that is part of the human microbiota. It is most abundant on the skin, in the respiratory system and in the human digestive tract. Also, *Staphylococcus aureus* contributes to human infections and has a high mortality rate. Both of these bacterial species produce biofilm, a pathogenic factor increasing their resistance to antibiotics. For this reason, we are looking for new substances that can neutralize bacterial cells. One of the best-known substances with such effects are silver nanoparticles. They exhibited antibacterial and antibiofilm formation activity that depended on their size, shape and the concentration used. In this review, we presented the data related to the use of silver nanoparticles in counteracting bacterial growth and biofilm formation published in scientific papers between 2017 and 2021. Based on the review of experimental results, the properties of nanoparticles prompt the expansion of research on their activity.

## 1. Introduction

The genus *Staphylococcus* includes many species that are physiological and pathogenic flora for humans [1,2]. Staphylococci accounted for nearly 75% of all isolates from orthopaedic patients with infections [3]. The most prominent and the most common species is *Staphylococcus aureus*. It contributes to various infections characterized by high mortality, i.e., *S. aureus* was responsible for 18.9% of surgical site infections [4]. In addition, it is characterized by the ability to develop resistance mechanisms that make the treatment difficult [5]. According to this, in China, the proportion of hospital-acquired methicillin resistant *S. aureus* (MRSA) has reached 50.4% [6].

In addition to *S. aureus*, *S. epidermidis* is the second most frequently isolated species [7]. This microorganism, classified as Coagulase-Negative Staphylococci (CoNS), forms the microbiota of the skin, mucous membranes, and respiratory tract in humans [7,8]. It constitutes 65 to 90% of microorganisms isolated from the skin and mucous membranes, and at the same time causes nosocomial infections, i.e., bacteremia, perioperative infections or urinary tract infections [2,7,8,9].

**Figure 1:**
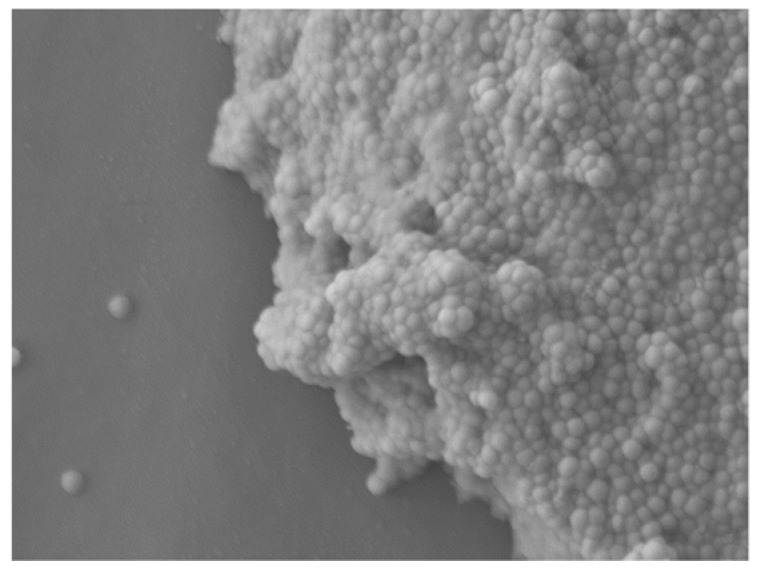
*S. epidermidis* biofilm visible under scanning electron microscope (×4300) FE-SEM 7600F (JEOL), equipped with “Cryo-SEM” (own source).

Both of these staphylococci species are associated with periprosthetic joint infections following surgery. This is due to their ability to produce biofilm - a pathogenic factor increasing the resistance of these microorganisms to the antibiotic therapy being applied [10]. The structure of the biofilm surrounding bacteria includes proteins, polysaccharides and extracellular DNA (Figure 1) [11]. The formation of biofilm in *S. epidermidis* is associated with the production of polysaccharide intercellular adhesin (PIA) encoded by genes in the *icaADBC* operon [9]. There are a limited number of drugs that enable the eradication of infections caused by bacteria producing biofilm [5]. In addition, some substances used in subinhibitory concentrations can stimulate the production of this virulence factor [12]. The growing resistance of biofilm-producing bacteria and the limited amount of substances that can be used in the case of infections with these microorganisms inspires the search for new therapeutic options [5].

The activity of plant substances, including monoterpenes, phenolic compounds and flavonoids, has been proven in this respect [13]. The antimicrobial and antibiofilm activity of nanoparticles is also of great importance [14]. Such properties have been proven in the case of titanium dioxide nanoparticles, zinc oxide (ZnO), tin oxide (SnO_2_), and cerium oxide (CeO_2_) nanoparticles [14,15]. In addition, gold nanoparticles (AuNPs) exhibit antibacterial and antibiofilm formation activity. Otari et al. [16] carried out the rapid synthesis of gold nanoparticles in an alginate polymer. The AuNPs were shown to have an antimicrobial activity against pathogenic bacteria. Among the metal compounds with antimicrobial activity, the most common are silver compounds. Their use has increased since the last century. Apart from their use in the textile industry and food packaging, their antibacterial activity has also been proven. Silver nanoparticles (AgNPs) have significantly higher antibacterial properties in comparison with silver ions. The bactericidal effect of colloidal nanosilver has been confirmed against Gram-negative bacteria and drug-resistant MRSA strains [17]. Such a broad spectrum of activity against morphologically and metabolically different microorganisms and strong antibacterial activity seems to be associated with a multidirectional range of antimicrobial activity [18].

Taking into account the above mentioned data, the aim of this review article is to show the antibacterial mechanism of nanosilver’s action that depends on their shape, size and concentration of the solutions used. This review is also focused on the various methods related to the obtaining of silver nanoparticles presented in the literature in the last five years, which allows us to indicate the most efficient methods for their synthesis. Moreover, it is possible to compare the antimicrobial activity of silver nanoparticles depending on the method of their synthesis or by their size and shape. We also indicate the possibility of combining nanosilver with other antibacterial compounds in order to increase their activity.

## 2. Mechanism of Action

**Figure 2:**
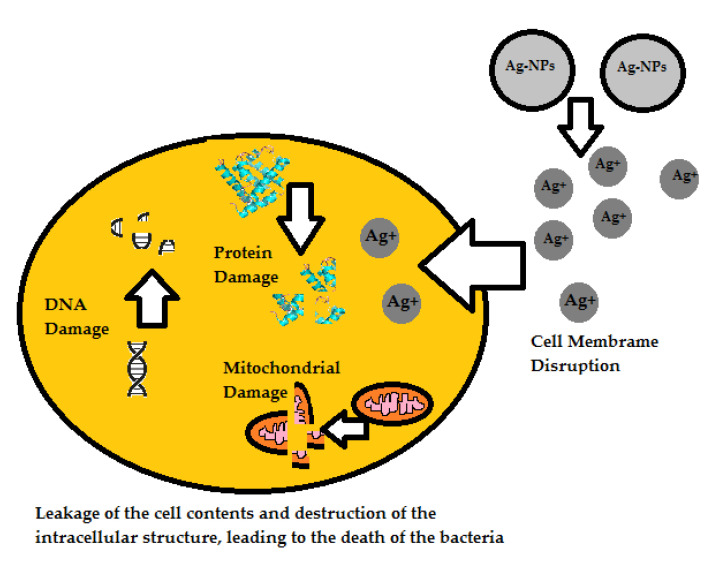
Mechanism of antimicrobial activity of silver nanoparticles.

The bactericidal activity, which is detrimental to the survival of bacterial cells, is caused by the release of silver ions from nanoparticles [19]. The mechanism of their action against staphylococci is the irreversible damage of bacterial cells by inhibiting the bacterial DNA replication, degradation of the bacterial cytoplasm membranes or the modification of intracellular adenosine-5′-triphosphate (ATP) levels (Figure 2) [18]. Silver ions have a high affinity for electron donating groups that are widely found in cell membranes or proteins such as the sulfhydryl, carbonyl, and phosphate groups. They can also bind to protein thiol groups, change their three-dimensional structure, and thus block active binding sites [19]. The special structure and multiple ways of contact with the bacterial cell membrane give them a unique way of counteracting the growth of bacteria [17].

Silver nanoparticles can damage the cell membrane through direct contact and can attack the respiratory chain [18,20]. Bacterial cells exposed to nanosilver particles are damaged by various mechanisms. One of them is damage of the peptidoglycan structure. It is present in the structure of the cell wall of Gram-positive and Gram-negative bacteria. The secondary structure of the amide groups belonging to the peptide chain is changed. The disappearance of sugar bonds and the release of muramic acid are also observed. This is probably one of the causes of the formation of “pits” in the cell wall. The impairment of the peptidoglycan structure may consequently result in the cell lysis by disturbing the osmotic balance of the cytoplasm [21].

AgNPs also damage lipoteichoic acids present in the cell wall of Gram-positive bacteria. The saccharide components of acetylglucosamine are broken down and d-alanine is damaged. Lipoteichoic acids are believed to be essential for the growth and metabolism of bacteria. For this reason, their complete degradation is likely to inhibit bacterial growth [21]. It has been proven that the pH and osmotic pressure of the solution can also increase due to the release of ions from the nanoparticles. In addition, silver nanoparticles affect phospholipids, including phosphatidylethanolamine, causing the loss of its amphiphilic character. This can cause the membranes to lose their barrier function, leading to cytoplasm leakage [19,20].

Other studies have reported on the ability of AgNPs to produce reactive oxygen species that destroy intracellular structures or their possibility to modulate bacterial signaling pathways. In the presence of nanosilver particles, superoxide and hydroxyl radicals are formed without the participation of cells. This abiotic generation of reactive oxygen species (ROS) is most likely the source of AgNPs attack on components of the cell envelope. Damage to the cell wall structure can lead to the entry of toxic silver particles into the bacteria and their accumulation in the bacterial inner membrane [20,21].

In turn, an increased production of superoxide radicals was observed in cells after exposure to AgNPs. Approximately three-fold higher ROS cell signals were also detected as compared to samples containing only cells (no silver). Some studies have also shown the increased expression of the *sodA* gene, which codes the superoxide dismutase (MnSOD) subunit after exposure to AgNPs. It has been shown that superoxide and hydroxyl radicals damage intracellular molecules. There is a peroxidation of lipids and damage of DNA, RNA and amino acids in proteins (especially cysteine and methionine). The antioxidant mechanisms of bacteria are defeated by the increased production of ROS, leading to the inhibition of growth and cell death [21].

## 3. Properties of Nanoparticles

The antimicrobial activity of nanosilver depends on many factors influencing the different effectiveness of the preparations being applied. The shape of the nanoparticles used is certainly important. Numerous studies have reported a difference in the activity of silver nanoparticles, depending on their shape. As indicated by Tanvir et al. [22], prismatic (truncated) silver nanoparticles with sharp peaks and edges with a size of about 20 nm showed better antibacterial activity as compared to spherical forms of similar diameter. This result was also confirmed in a study by Pal et al. [23]. The authors also indicated a higher reactivity of prismatic particles, in comparison with spherical particles. According to both teams, this difference might result from the construction of prismatic forms, which demonstrated a higher atomic density. It is also suggested that the planes of the prismatic forms might have lower surface tension. Therefore, a high reactivity of truncated nanoparticles as compared to spherical or rod-shaped particles was observed [23]. Also, Alshareef et al. [24] found the influence of nanosilver shape on its antimicrobial effectiveness. Truncated octahedral nanoparticles were shown to be bactericidal against *E. coli*. In turn, the spherical nanosilver had a bacteriostatic effect. It has been suggested that the bactericidal activity was due to their geometric structure, larger surface area, and higher surface energies in comparison with spherical nanoparticles [24,25]. In a study by El Zahry et al. [26], hexagonal silver nanoparticles were found to be more toxic than spherical and triangular nanoparticles with rounded edges (Table 1). This activity was claimed to be due to the high ability to penetrate the bacterial cell wall, leading to cell death. 

Furthermore, the use of different concentrations of silver nanoparticles caused a different antibacterial effect. In a study by Platania et al. [27], a decrease in the number of *S. aureus* and *S. epidermidis* was observed along with an increase in the concentration of nanosilver with a specific particle size.

Many studies, in addition to the very satisfactory effect of silver nanoparticles as a single antibacterial compound, indicated better effects if they were used in combination with other substances, e.g., tryptophan [19,28]. A study by Leng et al. [19] showed that bonding silver nanoparticles with zinc nanoparticles turned out to be more effective as compared to using them separately. In another study, the coating of silver nanoparticles with poly-L-arginine was used to enhance their interaction with cells [22]. The synergistic effect was also demonstrated by tobramycin in combination with silver nanoparticles with a particle size of 10–60 nm [29]. In turn, Yu et al. [30] revealed that silver nanoparticles showed synergistic and additive effects with quorum sensing inhibitors, and antagonistic effects with sulfonamides additives. These results confirmed the improved action of AgNPs in combination with antibiotics.

## 4. Synthesis of Nanoparticles

The widespread interest in silver nanoparticles is also related to their synthesis. This can be carried out using chemical, physical and biological methods [31]. Disadvantages of physical and chemical methods include the use of expensive equipment, high energy consumption, and low production efficiency [32]. These methods often use highly reactive chemical compounds (reducing agents) with a potential biological hazard, such as hydrazine, sodium borohydride (NaBH_4_), dimethylformamide (DMF), and cetyltrimethylammonium bromide (CTAB) [33]. In the process of chemical synthesis, ethylene glycol serves as a reducing agent, AgNO_3_ as a nanosilver precursor, and polyvinylpyrrolidone (PVP) as a stabilizing agent. This method is more frequently used in the production of AgNPs [34].

We analyzed the data published in 2017–2021 that presented the most current use of silver nanoparticles in the antimicrobial activity against staphylococci. Table 2 presents information about silver nanoparticles synthesized with compounds of various origins and their effect on the *S. aureus* and *S. epidermidis* strains.

In recent years, many studies have focused on the potential of silver nanoparticle production from natural compounds [59]. Biologically synthesized nanoparticles are less toxic and more environmentally friendly [31]. In the work presented by Yuan et al. [59], quercitin, a natural compound from the flavonol family, was used for the production of nanosilver with a size of 10 to 50 nm (average size: approx. 11 nm). In turn, Gurunathan et al. [60] synthesized silver nanoparticles from *Allophylus cobbe* leaf extract. The nanomolecules had uniformly spherical shapes and their average size fluctuated around 5 nm. *Areca catechu* extract has also been used for the efficient synthesis of AgNPs. Nanoparticles produced by this method showed effective antibacterial activity [61]. Also, Otari et al. [62] biosynthesized silver nanoparticles decorated silica nanoparticles. A novel green tea leaf-based method was developed by this group. The product displayed antimicrobial activity against the *S. aureus* strain. The presence of compounds such as terpenoids, flavonoids or amino acids in extracts from various parts of plants makes it possible to use them as silver ion reducing agents. In this way, silver nanoparticles are formed and the process is faster, as compared to the methods of synthesis involving bacteria or fungi [63]. In turn, the synthesis of silver nanoparticles using bacteria can be achieved with the use of biomass, culture supernatant and cell free extract (CFE) [31,64,65]. Nadhe et al. [31] performed the synthesis of silver nanoparticles with the CFE from 14 isolates of *Acinetobacter* spp. The method was optimized by using different temperatures, concentrations of AgNO_3_ solution, and growth time of bacterial cultures. Silver nanoparticles with the size in a range from 10 to 60 nm were obtained. They showed dose-dependent antifungal and antibiofilm formation activity. As the authors emphasized, the synthesis of AgNPs using bacteria is more advantageous than the synthesis using plants. This is due to the ability to control AgNPs synthesis conditions and the easy growth of the bacterial culture [31].

Table 3 presents information about silver nanoparticles synthesized from natural raw materials depending on their size, acquisition methods and effect on the *S. aureus* and *S. epidermidis* strains.

## 5. Application of Nanoparticles

The antimicrobial activity of silver nanoparticles allows them to be used in a variety of various everyday products. Gels, medical equipment and dressings with the addition of nanosilver are widely used. Recently, the use of nanosilver active against multidrug-resistant (MDR) bacteria in the form of a hydrogel or in a topical, oral or even intravenous form has been proposed. Interestingly, the preparations used were non-cytotoxic to human leukemic cells, cancer cells, and mouse fibroblasts at the concentrations used. The possible use of nanosilver as an additive to detergents or fibers used for the production of clothing, in order to enhance their antibacterial effect, was also indicated. However, few products have been tested for high-resistant bacteria [63].

Given the growing use in medicinal products, the release of nanosilver into the environment and its impact on it are of increasing importance. One of the most widely known lesions caused by nanosilver is argyria. Moreover, people applying nanosilver developed bluish-colored skin. The mechanism of these changes has not been fully explained. In addition, nanosilver undergoes changes in environmental and biological media. There is a loss of surface coating agent due to nanosilver dispersion. The nanosilver particles become partially uncoated due to the lack of suitable coating agents. The agglomeration of nanosilver was also observed. This phenomenon occurs due to the displacement of the coating agents by other particles and the loss of the stability of nanosilver. Finally, nanosilver particles can oxidize the surface and release silver ions. Other studies have also indicated that the transformation of nanosilver in the environment is strongly influenced by lighting conditions, the concentration of sulphur ions, dissolved oxygen, biological macromolecules (DNA and protein), and other organic compounds with a strong affinity for silver [80].

The use of these nanomolecules in vivo may be impaired by various inhibitory mechanisms. In addition, silver nanoparticles may cause immunotoxicity, cytotoxicity and genotoxicity to human cells. The foregoing observations suggest that local application seems to be the most appropriate [81]. 

With such a wide use of nanosilver, there is a concern about the possibility of bacterial resistance to the nanomaterials used. Resistance to silver ions due to the presence of a plasmid, the carrier of the *silE* resistance gene, has been known for a long time. There is also endogenous (mutational) resistance to silver, e.g., in the *rpoB* gene. Most of these studies look at silver ion resistance. In the case of silver nanoparticles, ion preservation is one of the ways in which they work [63]. There are also mutations associated with AgNPs resistance and those that can protect against both AgNPs and ionic silver. This is where AgNPs/ion silver cross-resistance occurs. These mutations were detected after exposure to nanosilver, which means that the spread of resistance traits will continue even after silver use is discontinued. Such hereditary resistance to nanosilver in pathogens warns against the careless use of these antimicrobials [82].

There is a great need for the study of new antimicrobial substances due to the resistance of bacteria increasing at a constant rate. First of all, we look for substances with antibacterial activity that affects many species of bacteria. It is also important to inhibit the bacterial biofilm, which makes the quick eradication of infection more difficult. Nanoparticles undoubtedly demonstrate the required properties. They can fight superbacteria due to their unique physicochemical properties. The most known silver nanoparticles have been proven to have strong antibacterial properties. An additional advantage of nanoparticles is the phenomenon of mutual overlapping of their antibacterial mechanisms. This reduces the risk of emerging bacterial resistance [83].

Table 4 presents information about silver nanoparticles synthesized with the participation of living organisms and their effect on *S. aureus* and *S. epidermidis* strains.

## 6. Discussion

As can be seen in the tables presented above, silver nanoparticles showed differentiated antimicrobial efficacy against staphylococci. The growth inhibition of both bacterial species by the silver nanoparticles used was in a range from about 60 to even as high as 100%. In the case of *S. epidermidis* strains, the size of the zones of inhibition for the silver nanoparticles ranged from 4.5 to 39 mm, while foror *S. aureus* strains it was 4–30.12 mm. The minimal inhibitory concentration (MIC) of *S. epidermidis* microorganisms ranged from 0.19 to 4000 µg/mL (median: 12.5 µg/mL; Q_1_ = 5 µg/mL; Q_3_ = 94 µg/mL). Similarly, for *S. aureus* the MIC range was from 1.14 to 4000 µg/mL (median: 39 µg/mL; Q_1_ = 15.63 µg/mL; Q_3_ = 125 µg/mL). Compared to Gram-negative bacteria, there was a much higher MIC value found for the silver nanoparticles preparations. This is due to the higher resistance of Gram-positive bacteria, conditioned by a thicker layer of peptidoglycan in their cell wall. The ability to produce biofilm also increases the resistance of the preparations and decreases the MIC value [42]. The presented works highlighted the killing mechanism of silver nanoparticles. It followed through the degradation of bacterial cell membranes upon interaction and incubation [67].

The studies of several teams focused on the diversified effect of silver nanoparticles on bacteria and their biofilm, depending on their size [17,41]. For spherical particles, as the particle size decreases, the surface area to volume ratio increases significantly. The result of such a phenomenon is the greater activity of nanoparticles with a smaller diameter [21,41]. The mentioned silver nanoparticles were divided into two groups. The first group consisted of nanoparticles of sizes ranging from 0 to 20 nm. The second group includes nanoparticles larger than 20 nm. When the MIC values were compared for both groups, there was an approximate 2.5-fold increase in MIC in the second group for *S. aureus* and *S. epidermidis*. In the case of *S. aureus*, the median in the first group was 19.5 µg/mL, while in the second group the median was 50 µg/mL. For *S. epidermidis* the results are 6.25 µg/mL and 15 µg/mL, respectively. Additionally, silver nanoparticles have a much higher surface-to-volume ratio as compared to other materials. This facilitates interaction with the surfaces of microorganisms, which determines a higher antimicrobial activity [21,89]. The differences in the effectiveness of nanoparticles were also shown, depending on the test temperature (28 °C, 37 °C) and the time of exposure of bacteria [17].

## 7. Conclusions

In this review, we summarized the current knowledge about the substances used in the production of silver nanoparticles. We also considered the antimicrobial and antibiofilm activity of the nanoparticles, with a particular focus on the effects on staphylococci.

As indicated above, nanoparticles can be synthesized with the use of chemicals, natural extracts or even living organisms. Many researchers used different methods for their production. This proves the great interest in the possibilities of their application. As shown in the tables above, the nanosilver particles show antibacterial and antibiofilm formation effects depending on their size, shape, and concentration used. The low MIC values obtained in many cases prove the good antistaphylococcal activity of nanosilver. Based on the review of experimental results, the properties of nanoparticles prompt the expansion of research on their activity. They also offer hope for a wider clinical application of nanosilver in the future. 

## Figures and Tables

**Table 1:** Antibacterial activity of different forms of AgNPs.

AgNPs	PEG AgNPs	Citrate AgNPs	Borohydride AgNPs	Deionized H_2_O (Negative Control)
Shape	hexagonal	spherical	triangular	-
Inhibition zone size (cm) ±SD	2.45 ± 0.24	2.02 ± 0.55	0.08 ± 0.14	0.00 ± 0.00

PEG (*Polyethylene Glycol*).

**Table 2:** Silver nanoparticles synthesized with compounds of various origins used against staphylococci.

Synthesis or Source	Size [nm]	Bacterial Strain	Concentrations Used and Results	Ref.
Lignin-Silver nanoparticles	20	*S. epidermidis* MDR	MIC = 10 µg/mL; MBC = 10 µg/mL	[35]
		*S. aureus* MDR	MIC = 10 µg/mL; MBC = 10 µg/mL	
		*S. aureus* ATCC 700788	MIC = 5 µg/mL; MBC = 10 µg/mL	
Mercaptosuccinic acid (MSA)-coated silver nanoparticles	2.98	*S. aureus*	ZOI = 8 mm; viability of bacterial cells reduced to 95%; significant biofilm disruption and elimination with 50 μg/g AgNPs loaded gel	[36]
		*S. epidermidis*	ZOI = 13 mm; viability of bacterial cells reduced to >96%; significant biofilm disruption and elimination with 50 μg/g AgNPs loaded gel	
Luteolin tetraphosphate derived silver nanoparticles	9; 21	*S. epidermidis* ATCC	viability below 70% for concentrations from 1 to 15 µM	[37]
The modified methoxypolyethylene glycol bishydrazino-s-triazine silver nanoparticles (mPEGTH2-AgNPs 2:1)	7–10	*S. epidermidis* ATCC 12228	MIC = 0.094 mg/mL; MBC = 0.188 mg/mL	[38]
Sulfonated polystyrene beads with embedded silver nanoparticles (PSSAg)	5	*S. aureus* ATCC 29213	MIC = 1.14 µg/mL; MBIC = 3.04 µg/mL	[39]
		*S. epidermidis* ATCC 12228	MIC = 0.76 µg/mL; MBIC = 0.76 µg/mL	
Composite hydrogel (CoHy) of RSF stabilized with Carboxymethyl Cellulose-Na (CMC-Na) and loaded with silver nanoparticles	92	*S. epidermidis* ATCC 35984	ZOI = ~4.5 mm	[40]
		*S. aureus* ATCC 29737	ZOI = ~6.0 mm	
		MRSA ATCC 33591	ZOI = ~4.0 mm	
Silver nanoparticles	10	*S. epidermidis* ATCC 12228	MIC = 3 µg/mL	[41]
		*S. epidermidis* ATCC 35983	MIC = 4 µg/mL	
		*S. epidermidis* ATCC 35984	MIC = 5 µg/mL	
Silver nanoparticles produced with AgF (pAgNP-F)	7.0–7.2	*S. epidermidis* RP62A	MIC = 250 µM	[42]
Nanopowder of silver nanoparticles (AgNPs) coated with polyvinyl pyrrolidone (PVP)	20–30	*S. aureus* ATCC 6538	ZOI = 11.5 mm; MIC = 4000 µg/mL	[43]
		*S. epidermidis* ATCC 35984	ZOI = 10.6 mm; MIC = 4000 µg/mL	
Silver nanoparticles synthesized with the zirconium phosphate glycine-N,N-bismethylphosphonate	4.5	*S. epidermidis* ATCC 35984	ZOI = 6.2 mm	[44]
Tryptophan Silver nanoparticles (2:1)	~13.4	*S. epidermidis*	100% inhibition of growth after 24 h incubation; inhibition of biofilm formation of 20.0% to 40.2%	[28]
		*S. aureus*	100% inhibition of growth after 24 h incubation; inhibition of biofilm formation of 67.2% to 71.8 %	
Silver nanoparticles/saponite nanocomposites obtained from initial silver ion concentration of 500 mg/L	2–4	*S. aureus* ATCC 11632	ZOI = 23.50 ± 0.87 mm	[45]
		*S. epidermidis*	ZOI = 30.67 ± 0.58 mm	
Porphyrin-silver nanoparticles with LED illumination	5 to 30 range, the majority of silver nanoparticles had a size of about 10	*S. epidermidis* DBM 3179	the proposed AgNPs-porphyrin conjugate show pronounced antibacterial activity, which exceeds the activity of each separated antibacterial agents; LED illumination enhances the activity against the *S. epidermidis* strain	[46]
Powder preparation containing 0.03–0.05 wt.% silver nanoparticles	40–50	10 MRSE strains	microbial contamination of wounds decreased by 100 or more times in comparison the bacterial load before treatment	[47]
Titanate Nanotubes Modified with Silver Nanoparticles	1–10	*S. epidermidis* ATCC 49461	the samples containing higher Ag loading were more active than samples with low silver amount	[48]
Graphene Oxide-Silver Nanoparticles mixture-coated polyurethane foil	251 ± 10	*S. aureus* ATCC 25923	79.6% growth inhibition	[49]
		*S. epidermidis* ATCC 14990	76.5% growth inhibition	
Zero-valent nano-silver/TiO_2_-chitosan composite	6.69–8.84	*S. epidermidis*	ZOI = 8.56 mm for 2 wt%; critical concentrations: 7.57 µg Ag/mm^3^ for 0.5 wt%; 2.96 µg Ag/mm^3^ for 1 wt%; 1.51 µg Ag/mm^3^ for 2 wt%	[50]
Microbeads with silver nanoparticles, obtained by reducing silver ions with ascorbic acid in Chitlac solution	270 ± 40	*S. epidermidis* ATCC 12228	Growth inhibition of more than four orders of magnitude	[51]
		*S. aureus* ATCC 25923	Growth inhibition of nearly four orders of magnitude	
The dendrimer-encapsulated Ag nanoparticles (G5-AgNPs) at available Ag–G5 feeding molar ratio of 30:1	3.33	*S. aureus* USA300	MIC = 128 µg/mL	[52]
The dendrimer-encapsulated Ag nanoparticles (G5-AgNPs) at available Ag–G5 feeding molar ratio 30:1 reacted with oxidized dextran (Dex-CHO)-Dex-G5–Ag (30) hydrogel	3.33	*S. epidermidis* 1457	99.8% inhibition of growth at the concentration 16 µg/mL	[53]
		*S. aureus* USA300	99.9% inhibition of growth at the concentration 16 µg/mL	
Silver-decorated submicron-sized biodegradable poly (DL-lactide-coglycolide) nanoparticles (Ag PLGA nanoparticles)	299.6	*S. epidermidis* ATCC 14990T	38.5% viability of bacteria after treatment with Ag PLGA nanoparticles; viable colony counts for the biofilm decreased from 6.4 × 10^7^ CFU/mL to 1.4 × 10^7^ CFU/mL (more than four orders of magnitude); viable colony count for the bacterial cells after various treatment for 2 h decreased from 6.4 × 10^6^ CFU/mL to 3.7 CFU/mL (1.7 million times less)	[54]
Silver nanoparticles synthesized and coated with pectin	8.0	*S. epidermidis* RP62A	MIC = 500 µM	[55]
Phytosynthesized silver nanoparticles	15–35; approximately 27	*S. epidermidis*	BE = 3.36 (after 18 h of contact, calculated by growing bacterial suspensions and count viable cells using a digital colony counter)	[56]
		*S. aureus*	BE = 3.60 (after 18 h of contact, calculated by growing bacterial suspensions and count viable cells using a digital colony counter)	
Silver nanoparticles synthesized and supported by TiO_2_ powder (P25) using heat-treatment temperature 200 °C	3.02–5.74	*S. epidermidis* BCRC 11030	ZOI = 4.6 mm; 7.4 mm; 10.0 mm; 16.5 mm; 13.7 mm in Ag weight percentage: 0.5; 1; 2; 5; 10 wt%, respectively	[57]
Carboxy methyl cellulose stabilized silver nanoparticles	5–15	*S. aureus* MTCC 3160	ZOI = 28.23 mm; MIC = 60 µg/mL; MBC = 60 µg/mL; MBC/MIC ratio = 1.00	[58]
		*S. epidermidis* MTCC 435	ZOI = 25.03 mm; MIC = 70 µg/mL; MBC = 70 µg/mL; MBC/MIC ratio = 1.00	
Silver nanoparticles immobilized onto biomaterial	25	*S. aureus* MRSA ATCC 43300	100% growth reduction after 45 min of bacteria exposure to the action of silver nanoparticles at a concentration of 5 mM	[17]
		*S. epidermidis* ATCC 700567	100% growth reduction after 45 min of bacteria exposure to the action of silver nanoparticles at a concentration of 5 mM	

MIC (Minimum Inhibitory Concentration); MLC (Minimum Lethal Concentration); ZOI (Zone of Inhibition); MIC_90_ (Minimum Inhibitory Concentration Required to Inhibit the Growth of 90% Organism); MSSA (Methicillin-Sensitive Staphylococcus aureus); BE (Bactericidal Effect); LG (Lack of Growth); MRSA (Methicillin-Resistant Staphylococcus aureus); MRSE (Methicillin-Resistant Staphylococcus epidermidis); MBIC (Minimum Biofilm Inhibitory Concentration); MBC (Minimum Bactericidal Concentration); MDR (Multidrug-Resistant Bacteria)

**Table 3:** Silver nanoparticles synthesized from natural raw materials used against staphylococci.

Synthesis or Source	Size [nm]	Bacterial Strain	Concentrations used and Results	Ref.
Silver nanoparticles synthesized from the aqueous extract of *Cotyledon orbiculata*	106–137 ± 2	*S. aureus*	MIC = 20 µg/mL; MBC = 40 µg/mL	[66]
		*S. epidermidis*	MIC = 20 µg/mL; MBC = 20 µg/mL	
		MRSA	MIC = 40 µg/mL; MBC = 80 µg/mL	
Silver nanoparticles synthesized from aqueous ‘root extract’ of *Salvadora persica*	10–70	*S. epidermidis* ATCC 12228	MIC = 0.19 µg/mL; MBC = 0.39 µg/mL	[67]
Silver nanoparticles synthesized from *Populus ciliata* leaf extract	4	*S. epidermidis*	ZOI = 17.8 mm	[68]
Silver nanoparticles synthesized using *A. esculentus* flower extract	5.52–24.65; average 18.24	*S. aureus* ATCC 29213	MIC = 85 µg/mL; MBC = 90 µg/mL	[69]
		*S. epidermidis* MTCC 3615	MIC = 150 µg/mL; MBC = 165 µg/mL	
Silver nanoparticles synthesized using lyophilized hydroalcoholic extract of *S. cumini* seeds	36.25–77.01	*S. aureus* ATCC 25923	MIC = 125 µg/mL	[70]
		*S. epidermidis* ATCC 12228	MIC = 31.2 µg/mL	
Silver nanoparticles synthesized using lyophilized hydroalcoholic extract of *S. cumini* flowers	37.21–46.48	*S. aureus* ATCC 25923	MIC = 250 µg/mL	[70]
		*S. epidermidis* ATCC 12228	MIC = 250 µg/mL	
Silver nanoparticles synthesized with *Brassica oleracea*	20	*S. aureus* ATCC 6538	ZOI = 10.1 mm; MIC = 25 µg/mL	[71]
		*S. epidermidis* ATCC 12228	ZOI = 14.33 mm; MIC = 6.25 µg/mL	
Silver nanoparticles synthesized with the leaf infusion of *Dracocephalum kotschyi* Boiss	5–63; average 19.41	*S. epidermidis* ATCC 12228	ZOI = 13 mm; MIC = 15.63 µg/mL	[72]
		*S. aureus* ATCC 43300	ZOI = 18 mm; MIC = 15.63 µg/mL	
Silver nanoparticles synthesized by *P. chrysosphorium*	34–90	*S. epidermidis*	ZOI = 11 mm	[73]
		*S. aureus*	ZOI = 13 mm	
Biocompatible green-synthesized colloidal nanoparticles synthesized from *Olax nana* Wall. ex Benth. (family: *Olacaceae*) aqueous extract	26	*S. epidermidis*	MIC = 7.14 µg/mL	[74]
Silver nanoparticles synthesized with enzyme β-galactosidase from *Aspergillus oryzae*	12.89	*S. aureus*	MIC = 32 µg/mL; MBC = 64 µg/mL; over 80% inhibition of biofilm formation at the concentration 16 µg/mL	[75]
		*S. epidermidis*	MIC = 64 µg/mL; MBC = 128 µg/mL; the bacterial cells completely lose their count at a concentration of 32 µg/mL within 12 h; over 60% inhibition of biofilm formation at the concentration 16 µg/mL	
Silver nanoparticles synthesized from pomegranate fruit peel (*Punica granatum*)	20–40; average 26.95	*S. aureus* ATCC 29213	ZOI = 10 mm, 14 mm, 19 mm, 21 mm for the AgNPs concentration 25 µL, 50 µL, 75 µL, 100 µL, respectively	[76]
		*S. epidermidis* MTCC 3615	ZOI = 6 mm, 7 mm, 11 mm, 12 mm for the AgNPs concentration 25 µL, 50 µL, 75 µL, 100 µL, respectively	
Silver nanoparticles produced using the leaf extract of the plant *Arbutus unedo*	50–60	*S. epidermidis* isolate C5M6	MIC = 15 µg/mL MIC_50_ = 6.6 µg/mL MBC = 15 µg/mL	[77]
Silver nanoparticles produced using the leaf extract of the plant *Arbutus unedo*	40	*S. epidermidis* isolate C5M6	MIC = 15 µg/mL MIC_50_ = 6.3 µg/mL MBC = 15 µg/mL	[77]
Silver nanoparticles produced using gallic acid and apocynin from *Pelargonium endlicherianum* Fenzl. root extract	60	*S. epidermidis* ATCC 11228	MIC = 7.81 µg/mL; ∼81% of cell growth inhibition at concentration 62.5 µg/mL	[78]
Silver nanoparticles produced using gallic acid, apocynin and quercetin from *Pelargonium endlicherianum* Fenzl. root extract	25 and 80	*S. epidermidis* ATCC 11228	MIC = 6.25 µg/mL; ∼88% reduction of bacterial cell density at concentration 62.5 µg/mL	[78]
Silver nanoparticles synthesized using *Artemisia haussknechtii* leaf aqueous extract	10.69	*S. aureus* ATCC 43300	ZOI = 12 mm; 10 mm; 8 mm in concentrations: 0.1 M; 0.01 M; 0.001 M, respectively; MIC = 10 µg/mL; MBC = 60 µg/mL	[79]
		*S. epidermidis* ATCC 12258	ZOI = LG in concentrations: 0.1 M; 0.01 M; 0.001 MIC = 4 µg/mL; MBC = 20 µg/mL	

MIC (Minimum Inhibitory Concentration); MLC (Minimum Lethal Concentration); ZOI (Zone of Inhibition); MIC_90_ (Minimum Inhibitory Concentration Required to Inhibit the Growth of 90% Organism); MSSA (Methicillin-Sensitive Staphylococcus aureus); BE (Bactericidal Effect); LG (Lack of Growth); MRSA (Methicillin-Resistant Staphylococcus aureus); MRSE (Methicillin-Resistant Staphylococcus epidermidis); MBIC (Minimum Biofilm Inhibitory Concentration); MBC (Minimum Bactericidal Concentration)

**Table 4:** Silver nanoparticles synthesized with the participation of living organisms used against staphylococci.

Synthesis or Source	Size [nm]	Bacterial Strain	Concentrations Used and Results	Ref.
Silver nanoparticles obtained from *Lactobacillus bulgaricus*	30–100	*S. aureus*	ZOI = 15 mm	[84]
		*S. epidermidis*	ZOI = 17 mm	
Silver nanoparticles synthesized from *Bacillus subtilis*	3–20	*S. aureus* (MRSA)	ZOI = 30.12 mm; MIC = 230 µg/mL; MLC = 380 µg/mL	[85]
		*S. epidermidis*	ZOI = 39.0 mm; MIC = 180 µg/mL; MLC = 220 µg/mL	
Silver nanoparticles derived from marine *Streptomyces* sp. Al-Dhabi-87	20–50	*S. aureus* ATCC 29213	MIC = 0.039 mg/mL; 100% inhibition of bacterial growth	[86]
		*S. epidermidis* ATCC 12228	MIC = 1.25 mg/mL; 100% inhibition of bacterial growth	
		Multidrug Resistant *S. aureus* WC 25 V 880854	MIC = 0.039 mg/mL	
		Multidrug Resistant *S. aureus* V 552	MIC = 0.039 mg/mL	
		*S. aureus* ATCC 43300	MIC = 0.039 mg/mL	
		*S. aureus* TC 7692	MIC = 0.039 mg/mL	
Biologically obtained nanoparticles synthesized by *Actinomycetes* strain called CGG 11n	76.63	*S. aureus* ATCC 6338	ZOI = 7 mm; MIC = 25 µg/mL	[87]
		*S. epidermidis*	ZOI = 12 mm; MIC = 6.25 µg/mL	
Biologically obtained nanoparticles synthesized by *Actinomycetes* strain called CGG 11n functionalized with Ampicillin	95.04	*S. aureus* ATCC 6338	ZOI = 5 mm; MIC = 50 µg/mL	[87]
		*S. epidermidis*	ZOI = 12 mm; MIC = 6.25 µg/mL	
Silver nanoparticles synthesized by *Lactococcus lactis* 56 KY484989	5–50; average 19	*S. epidermidis* ATCC 49461	ZOI = 16 mm (in concentration 15 μg/mL); MIC_90_ = 3.13 µg/mL	[88]
		*S. aureus* MSSA ATCC 29213	ZOI = 12 mm (in concentration 15 μg/mL); MIC_90_ = 12.5 µg/mL	
		*S. aureus* ATCC 6338	ZOI = 14 mm (in concentration 15 μg/mL); MIC_90_ = 3.13 µg/mL	

MIC (Minimum Inhibitory Concentration); MLC (Minimum Lethal Concentration); ZOI (Zone of Inhibition); MIC_90_ (Minimum Inhibitory Concentration Required to Inhibit the Growth of 90% Organism); MSSA (Methicillin-Sensitive Staphylococcus aureus); BE (Bactericidal Effect); LG (Lack of Growth); MRSA (Methicillin-Resistant Staphylococcus aureus); MRSE (Methicillin-Resistant Staphylococcus epidermidis); MBIC (Minimum Biofilm Inhibitory Concentration); MBC (Minimum Bactericidal Concentration)
